# Protein Folding: Search for Basic Physical Models

**DOI:** 10.1100/tsw.2003.50

**Published:** 2003-07-30

**Authors:** Ivan Y. Torshin, Robert W. Harrison

**Affiliations:** Department of Biology, Georgia State University, Atlanta, Georgia 30303, USA

**Keywords:** protein folding, hydrophobic core, electrostatic interactions, folding core, folding nucleus, protein structure prediction, bioinformatics

## Abstract

How a unique three-dimensional structure is rapidly formed from the linear sequence of a polypeptide is one of the important questions in contemporary science. Apart from biological context of *in vivo* protein folding (which has been studied only for a few proteins), the roles of the fundamental physical forces in the *in vitro* folding remain largely unstudied. Despite a degree of success in using descriptions based on statistical and/or thermodynamic approaches, few of the current models explicitly include more basic physical forces (such as electrostatics and Van Der Waals forces). Moreover, the present-day models rarely take into account that the protein folding is, essentially, a rapid process that produces a highly specific architecture. This review considers several physical models that may provide more direct links between sequence and tertiary structure in terms of the physical forces. In particular, elaboration of such simple models is likely to produce extremely effective computational techniques with value for modern genomics.

## EXPERIMENTAL STUDIES AND THERMODYNAMIC ANALYSES THAT MATCH THE EXPERIMENT

Detailed experimental studies[1] have shown an amazing feature of protein folding as a physical process: formation of complex architectures is extremely fast and can occur even in a microsecond. In comparison to many other of the related cellular processes (for example, synthesis of the polypeptides or formation of protein-protein interfaces), native-like structures emerge during the folding of a polypeptide chain almost instantaneously. At the same time, proteins have highly specific and regular "folds" (spatial architectures)[2,3] and these specific architectures are likely to be related to specific biological functions[4].

An important early event in the folding process is nucleation[5]. This process does not imply "classic" nuclei (such as those potentially identified by hydrogen exchange experiments[6]), but rather short "diffused" sequence regions and separate residues. Cooperativity of the folding (in thermodynamics) and nucleation (in kinetics) can be reproduced by folding simulations (at least in the case of some small proteins[7]). Statistical analyses of the energy landscapes derived from native structures[8,9,10] allow prediction of the folding mechanisms. One of the important conclusions that follow from the thermodynamic descriptions (TD)[10] is that mechanisms of the protein folding could be intrinsically related to the topology of the native state (i.e., final state of the folding). Although being largely diffusional models[11] that do not explicitly incorporate more basic physical forces, such models can identify potential folding nuclei[8].

Often, these thermodynamic models incorporate the notion that the lowest-energy states correspond to the native structures of globular proteins[12]. This assumption is not always true. For example, in alpha-lytic protease the unfolded states have a lower equilibrium free energy than the native state[13,14]. The native state of this protein is metastable[15]; its apparent stability perhaps derives from a large kinetic barrier to unfolding. In other words, the observation of kinetic stability (which is related to nonequilibrium phenomena) shows that the equilibrium free energy is not always crucial for the protein folding.

Folding of alpha-lytic protease[13,14] is made possible by an attached folding catalyst, the "proregion", which is degraded on completion of folding thus leaving the protein trapped in the native state by a large kinetic barrier. The native state of a protein being less stable than a partially folded or misfolded state shatters long-standing prejudices about protein folding[15]. The kinetics of folding and unfolding processes cannot be measured under identical conditions but a thorough characterization of the denaturant dependence[13,14] has allowed estimates of the unfolding rates in the absence of the denaturant. Despite an unusual peculiarity of this folding system (alpha-lytic protease folds efficiently to its metastable conformation only with the assistance of its proregion[13]), the thermodynamic postulate of "folding towards an energy minimum" is clearly not applicable to this protein. It is also interesting to notice that conformational rigidity of alpha-lytic protease is much higher than has ever been observed for "thermodynamically stabilized" proteins[14]. In other words, a kinetic barrier does not imply a loosely packed structure but quite the opposite, actually. The alpha-lytic protease is not the only protein for which the native folded state is *not* the lowest energy conformation. The recombinant mouse prion protein can be folded either to a monomeric alpha-helical or oligomeric beta-sheet–rich isoform. Using circular dichroism spectroscopy and size-exclusion chromatography, it was shown that the beta-rich isoform (i.e., the misfolded form) is thermodynamically more stable than the native (correctly folded) alpha-helical isoform[16]. Free energy of folding of wild type subtilisin may be very small[15], thus the concept of the "folding funnel" (that implies that the native folded state is the lowest energy conformation) will not also be entirely applicable in this case.

## HYDROPHOBIC CORE MODEL

Structures of the bulk of the globular native proteins have hydrophobic interior and polar exterior. This observation of the native structures of proteins has suggested a global hydrophobic collapse to be an important stage in protein folding[17]. In the framework of the hydrophobic core model, the extended polypeptide chain is compacted in the manner of many small hydrophobic oil-drops that join into one large spot on water surface. However, additional energy terms may be important for protein folding[18]. The electrostatic interactions, long range (charged side chains, up to 15 Å) as well as short range (hydrogen bonding), may be not less important than the hydrophobic effect. In physical chemistry, the hydrophobic effect can be measured as the equilibrium partitioning of a nonpolar solute between aqueous and nonaqueous environments. Achievement of an equilibrium in such a system usually requires significant time. Consequently, the hydrophobic effect is likely to be more important for slow, long-term processes (like minor repacking of the already formed protein core, for example). However, electrostatic effects propagate at light speed and thus are likely to be important for rapid kinetics (a discussion of possible time scales of the electrostatic and hydrophobic effects is provided further).

There are other problems with "hydrophobic core" as a physically complete model. "Forces" that are referred to as driving protein folding (such as "burial of exposed hydrophobic groups"[15]) and those that oppose it (such as "loss of configurational entropy")[15] are not actual physical forces (such as electrostatics and Van Der Waals forces) but rather thermodynamic generalizations of the whole process. The term "hydrophobic effect" itself[19] is a thermodynamic approximation (another example of such an approximation would be Born cycle) and it implicitly includes both van der Waals and electrostatic interactions. Although the term "hydrophobic effect" does not explicitly represent any fundamental physical force (such as electrostatics), the closest analogy to "hydrophobic effect" in the realm of fundamental physical forces will be Van der Waals interactions. Van der Waals interactions are nonspecific and nondirectional, and the same two characteristics are also implied in the concept of "hydrophobic interactions".

"Hydrophobic interactions" are nonspecific, therefore any model that involves only hydrophobic interactions does not provide satisfactory explanations of the high fold specificity. Hydrophobicity-only patterns do not allow unequivocal recognition of protein folds[20]. The specific fold is more efficiently recognized using rather a binary pattern of hydrophobic and polar (including charged) residues. Such hydrophobic/hydrophilic binary patterns at least select unique fold topologies with higher accuracy[21]. Our results at CASP-5[22] also show that using charged-only or charged+hydrophobic patterns produces better results (in terms of correct fold recognition) than using hydrophobic-only patterns. Successful methods of *ab initio* protein modeling include potentials that mimic the physical forces (electrostatic and Van der Waals interactions) involved in protein folding[23]. Of course, the results of fold recognition do not provide any conclusive evidence for the roles of electrostatics and folding and can serve, at best, only as an indirect indication. Nevertheless, elaboration of simple physical models that take into account both the electrostatics and hydrophobicity (in terms of Van der Waals interactions) may allow finding more direct links between the sequence and the tertiary structure than it is possible with a classical thermodynamic approach.

## FOLDING IS A RAPID PROCESS

Most experimental studies on the dynamics of protein folding have been confined to time scales of 1 ms and longer[24]. The initial multiparallel steps in protein folding — such as intrachain diffusion and formation of alpha-helices, beta-hairpins, and loop structures — occur on the microsecond to nanosecond time scale[25]. At the same time, this complex and multiparallel process leads to formation of highly specific protein architectures. Cotranslational folding may be an effective way to reduce the complexity[26] (and also as suggested by an indirect evidence from structural studies[27]). However, cotranslational folding proceeds on the hundreds of milliseconds to second scale, while it is possible for a protein to fold in a microsecond[1]. The thermodynamic descriptions, based on the general theory of polymers, can be correlated with the experimental data[8,10,28] and the rapid folding of certain proteins thus can be described using an energy landscape in the shape of a folding funnel. Such descriptions alone, without the direct experimental data on protein folding, can predict little. They also are based on the assumption that the native state has the lowest energy, which is not always true[13,14,15,16], as it was already mentioned. Using such descriptions alone, it is hardly possible to analyze the roles of the two basic physical forces (electrostatics and Van der Waals) in the protein folding. The TD models have provided valuable explanations[8,9,10,12,28], however, the classical TD approach is only one of the many instruments in modern physics. It is limited to the thermodynamics of near-equilibrium states and the above examples of alpha-lytic protease, prion protein, and subtilisin might show that assuming near-equilibrium in protein folding is not always correct (alpha-lytic protease is stabilized by a large kinetic barrier to unfolding — apparently, a nonequilibrium state). Therefore, other appropriate theoretical tools, which can incorporate the rapid time scales, high specificity of protein architectures, and basic physical forces must be found and applied to study protein folding — the process that determines biological function of a protein.

## ELECTROSTATIC FORCES IN THE PROTEIN FOLDING PROCESS

Protein folding is a very complex physical process[29]. The polypeptide consists of thousands of atoms and, formally, millions of interatomic interactions are possible. How this complexity is solved in the actual process of folding is not well understood in fundamental physical terms: the physical forces. Thermodynamic descriptions can be successfully used to match experimental data[8], however, the concepts of "the native state" (which is a spatial structure) and "the folding" (which is a physical process) are *not* identical. Using the TD approach alone does not always prevent this confusion between a structure and a process. Modeling the folding kinetics by allowing only one residue to become ordered/disordered at a time[8,10] also disregards the essentially multiparallel nature of the folding process. These features of the TD descriptions suggest that further experimental and theoretical analyses of the roles of the physical forces in the protein folding are required.

Van der Waals forces are largely taken into account in the hydrophobic core model as was already mentioned. One of the main features of the hydrophobic core model is that it does not provide even a qualitative explanation for the rapid selection of a highly specific architecture. This feature is intrinsic to the hydrophobic core-only model and correlates with Van der Waals interactions (which are the primary physical force important for a hydrophobic effect), being short-range and nonspecific. Therefore, an investigation into the physical models that take into account some specific long-range interactions may give a clue for the specificity and the rapidity of the folding process. Electrostatic interactions can be short range (hydrogen bonding, for example, is a specific type of short-range electrostatic interaction that is important for secondary and super-secondary structure formation) and long range. Electrostatic interactions are more specific than Van der Waals interactions: charges of one sign form a repulsive, while charges of the opposite signs form an attractive interaction. Although the roles of the electrostatic interactions in protein folding are not well studied[30,31], there is growing experimental and theoretical evidence of the importance of electrostatics in protein folding[32,33]. Roles of electrostatic interactions in protein folding have been variously assessed from being of little importance[31] to being one of the most important factors determining the secondary structure[34]. Optimization of the electrostatic interactions in different proteins suggests that one of the roles of the electrostatic forces in the protein folding may be the rejection of the repulsive contacts[35].

## COMPARATIVE TIME SCALES OF ELECTROSTATIC AND HYDROPHOBIC EFFECTS

Any change in protein conformation requires rearrangement of the water structure and thus is limited by the relaxation time of water. The relaxation time of water can be determined experimentally; for example, using nonlinear optical Kerr effect microscope[36]. This approach allows registration of ultra-fast diffusive motions of intracellular water molecules. Although average relaxation times of water inside a cell and of pure water differ (1.4 and 0.8 ps, respectively, that is, the viscosity of the cell liquid is about 2 times higher than that of pure water at room temperature), both values are of order of 1 ps[36]. The relaxation time of water puts certain time constraints on any conformational changes of a macromolecule, independently of what physical force has induced the conformational change. It should be remembered, though, that variations of electrostatic field propagate at light speed; thus, the relaxation time of water describes *the minimal rate* of the propagation of the long-range electrostatic effects in proteins in water. However, for any kind of a "hydrophobic effect" (which is a clustering of hydrophobic groups after their escape from contact with the solvent; thus "hydrophobic effect" necessarily implies rearrangement of the water structure), the relaxation time of water describes *the maximal rate* of propagation of a "hydrophobic effect" in proteins.

Another important difference between polar (i.e., involved in electrostatic interactions) and hydrophobic surfaces is the structuring of water around the surfaces. It is commonly assumed that essentially all water in cells and around macromolecules has the same ideal motional and colligative properties as does the "bulk" pure water in the liquid state. However, water molecules adjacent to hydrophobic surfaces form pentagonal structural arrays (summarized in [37]). Experimental study of water-solute interactions in solutions of individual amino acids, peptides, and proteins shows that this model of solute-induced water structuring near hydrophobic surfaces gives excellent correlation with the experimental data[37]. Independently proposed concepts of "low density water" and "high density water"[38] are equivalent to the terms of "structured water" and "destructured water"[37]. Physically, water molecules adjacent to polar surfaces are dielectrically aligned towards the polar surface and thus are not in optimal orientation for hydrogen bonding with one another. Therefore, they do not form as many hydrogen bonds with one another as the water molecules over hydrophobic surfaces. Pentagonal arrangement of water molecules around hydrophobic patches was directly observed in structures of crambin at ultra-high resolution (0.54 Å)[39] and in lysozyme at high resolution[40]. The rearrangement of water accompanies any change in protein conformation. A pentagonal water arrangement around hydrophobic surfaces (the surfaces directly related to any kinds of "hydrophobic effects") contains more solvent-solvent hydrogen bonds and thus will require more time and energy to be restructured. As the water around the charged/polar groups is less structured, the rearrangement of water will take less time for polar groups. Moreover, the electrical field of a charged side chain will also radially orient the water molecules at somewhat greater distances from the charge and this longer-range destructuring of solvent-solvent hydrogen bonding can also contribute to faster propagation of conformational changes induced by the charged groups (i.e., due to electrostatic interactions).

There are other important differences between a "hydrophobic effect" (which can be described in terms of Van der Waals forces) and electrostatic interaction; these differences will further drive apart the relative time scales of the two processes. Firstly, hydrophobic effect, as any stochastic process, would be intrinsically slower. Secondly, "hydrophobic interaction" is short ranged, i.e., changes in conformation cannot be coordinated over distances larger than 3–5 Å. Electrostatic interactions are long range (12–15 Å). This was observed experimentally in a protein[41]. In molecular dynamics simulations of nucleic acids and proteins, an accurate treatment of the long-range electrostatic interactions is crucial for achieving stable nanosecond trajectories[42,43] and restricting long-range electrostatics; even a 14-Å limit is still too short a cutoff[44]. Thirdly, hydrophobic-hydrophobic interactions are nonspecific. Any pair of hydrophobic side chains always forms a weak attractive interaction with almost no significant geometrical restraints put on the possible configurations. Electrostatic interactions are more specific. Side chains of one charge sign repulse while side chains of opposite charge signs attract each other. Thus, the number of possible conformations of the molecule is less than it would be in the framework of hydrophobic-core only model. As the number of possible conformations is less, the total time of the "conformational search" will also be less for a system with electrostatically interacting groups. These multiple factors (structuring of the water, stochasticity, specificity or nonspecificity of interactions and their range) make difficult a quantitative *a priori* assessment of the relative time scales of the electrostatics and the hydrophobicity in the folding. However, it can be said that the conformational changes induced by the electrostatic interactions are likely to propagate at the level of the water relaxation time, which is ~1 ps (or even less than 1 ps because of destructuring of the water-water hydrogen bonding around the charged/polar groups, as already mentioned above). However, the rate of propagation of conformational changes due to hydrophobic interactions will be lower, perhaps several times or an order of magnitude (say, ~10 ps), although evidence on the actual differences is still to be found. Taking the time scale of folding of a protein to be ~1 μs[1], apparently, more of ~1 ps "steps" (induced by electrostatics, structurally specific interaction) can happen during a microsecond, while less of ~10 ps steps (induced by hydrophobicity, structurally nonspecific interaction) can be made during the microsecond. Therefore, more rapid and more specific electrostatic interactions can, in principle, dominate the fast-folding kinetics. The higher specificity of electrostatic interactions, whether short (as hydrogen bonding) or long range, can also resolve imaginary contradictions between various assessments of the role of electrostatics in protein folding (like a contradiction between the statements "of little importance"[31] and "most important factor"[34]).

## DEVELOPING NEW MODELS

Investigation into the new physical models of folding may require estimating the relative roles of the Van Der Waals and the electrostatic interactions. Mimicking the physical forces involved in protein folding[22] leads to more accurate predictions of protein structure. Several new models of folding show promise in further elucidation of the relative roles of the hydrophobic and the electrostatic interactions, as well as in determining more direct relationships between the sequence and the structure.

Hydrophobic collapse may be global (when the general architecture of a protein is formed) and local (collapse of a short sequence fragment). The local hydrophobic collapse depends on a much shorter sequence and thus will proceed more rapidly than the global one. Analysis of protein sequences of 23 fully sequenced bacterial genomes shows that protein structure can be viewed as a compact linear array of closed loops of 25–30 amino acid residues[45]. As the end-to-end contacts in the loops are likely to be hydrophobic[45], this opens a new perspective to understand protein folding as consecutive looping of the polypeptide chain with the loops ending at hydrophobic nuclei[45]. At least, this model correlates well with the biologically established notion of the cotranslational protein folding[23,45] and does not contradict the emerging understanding that protein folding is essentially a rapid process (thus, there is no time for a random search of the native architecture). Proteins often contain specific short sequences that may play a role as nucleation centers in protein folding[46]. Helix signal hypothesis[47] is also related to the local collapse, which may be both hydrophobic and short-range electrostatic[47].

Thermodynamic and experimental studies suggest that it should be possible to describe the fundamental physics of the folding process using relatively low-resolution models[10]. One of such low-resolution models is the charge centers (CC) model[33]. At low resolutions (for example, when considering a protein inside the cell), the electrostatic field of the whole molecule can be described in terms of charge center(s). The positive and negative charge centers characterize the general distribution of the positive and negative residues in a protein molecule. This model has no adjustable parameters and the CCs are calculated solely on the basis of the spatial structure using a simple nonempiric formula[33]. Several important implications of the CC model[33] are summarized as follows:

1. There seems to be a semi-quantitative correlation linking the distribution of the charged residues in the sequence and their distribution in the native (tertiary) structure. The constant in this correlation is specific for a certain "fold" type (alpha, a+b, a/b, and beta).
2. Short hydrophobic sequences in the framework of the CC model are likely to be involved in the local hydrophobic collapse[33], and this has been directly confirmed by experimental data at least in one case[48] and, in part, by genome-wide analysis of "hydrophobic loops"[45].
3. The analyses based on the CC model suggest the following general order of events in folding: local hydrophobic nucleation, electrostatic collapse of the core, global hydrophobic collapse, and slow annealing to the native state[33]. This scheme agrees with the experimental data for CI2[48].
4. The CC model allows the prediction of the residues of the hydrogen exchange core with relatively high accuracy (70–100%)[33]. The hydrogen exchange core is likely to be an important intermediate in the protein folding[6,49]. Moreover, an analysis of the radial distribution of the residues around the charge centers identifies the residues of the folding nuclei more precisely[48]. In terms of the identified residues, the CC model[33] produces results comparable to those produced by more detailed molecular dynamics analyses[50] and Monte Carlo simulations[51]. A recent experimental discovery of long-range interactions within a non-native protein[52] suggests that the significance of the hydrophobic collapse should not be overestimated and thus provides an additional support for the charge center model.

## COMPARISON OF BIOCHEMICAL AND STRUCTURAL DATA IN THE FRAME OF THE CHARGE CENTER MODEL: FOLDING OF CI2

Although the role of electrostatics in folding has not been studied in detail, electrostatic interactions may be particularly important for the folding of CI2 (chymotrypsin inhibitor 2)[53]. The charge center analysis[33] suggests four general stages of protein folding (presented in **Fig. 1**). Residues identified by the analytical procedure and those from the biochemical experiments are summarized in **Table 1**.

Let us consider folding of CI2 stage by stage. **Stage 1.** The analysis[33] suggests that *continuous hydrophobic sequences* (in CI2 there are 3 of them: 19–21 VIL; 29–35 IIVLPVG, and LFV 49–51) are likely to be the earliest folding events. Indeed, NMR experiments and molecular dynamics of CI2 unfolding[54] has shown "hydrophobic clustering" of the sequence 29–35 as well as 19–21 (which is part of the helix in CI2, **Fig. 1A**). Hydrophobic collapse would be significantly easier when hydrophobic residues are located sequentially, because there will be less of that so-called "random conformational search"[33]. **Stage 2.** *Nucleation* site for folding of the CI2 was suggested to include regions of the protein around the residues Ala-16, Leu-49, and Ile-57[54]. These residues belong to the residue clusters around the positive and negative charge centers (Table 1). The major event in the folding seems to be closing of the helix to the beta-sheet[54], which agrees with the charge centers being located between the helix and the beta-sheet (**Fig. 1B**). **Stage 3.** Hydrogen exchange cores are likely to be important intermediates in folding[6] and the residues of these cores can be identified using the CC model[33]. *Global hydrophobic collapse* would complete formation of such an intermediate core and also of the active/binding site (**Fig. 1C,** the binding loop is 54–64[55], residues 35–45 in PDB 1 ciq) The binding loop is located between the strands brought together in this stage. **Stage 4.** *Slow annealing* (**Fig. 1D**) is, perhaps, the longest stage in folding, and it would include minor repacking of the almost completely folded structure.

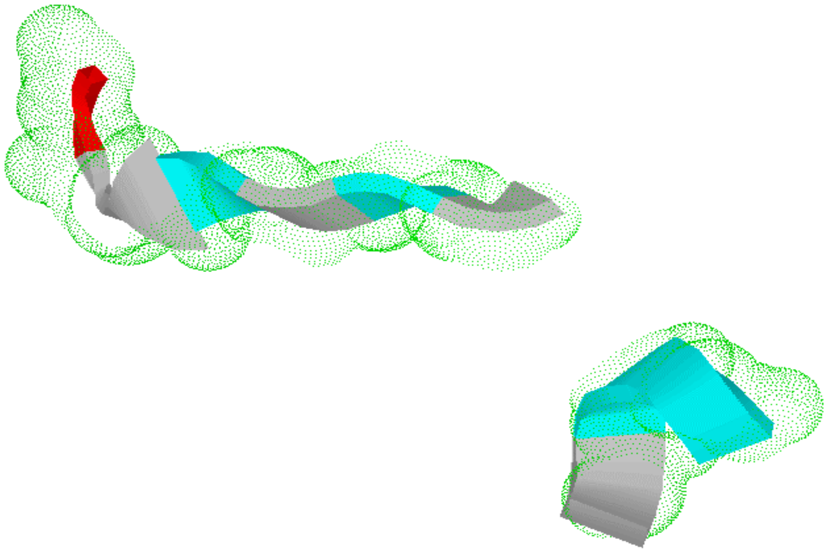

**FIGURE 1A**

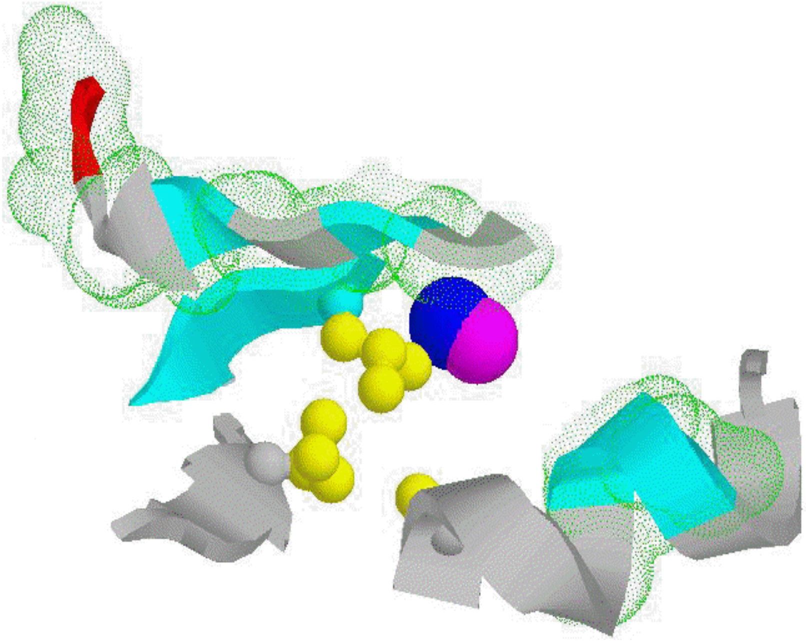

**FIGURE 1B**

**FIGURE 1.** The four-stage scenario for the folding of a protein, illustrated on the example of chymotrypsin inhibitor 2 (PDB 1 ciq), see text for the details on biochemistry of CI2 folding. (A) *Local hydrophobic collapse;* (B) formation of the residue cluster(s) around the charge centers, then *electrostatic collapse;* (C) *global hydrophobic collapse*, formation of the active/binding site; (D) *slow annealing* to the native state. HX (hydrogen exchange) residues (cyan), continuous hydrophobic sequences (green dots), residues of folding nucleus (yellow), charge centers (positive, blue sphere, negative, red), and the binding site (loop colored in red) are shown.

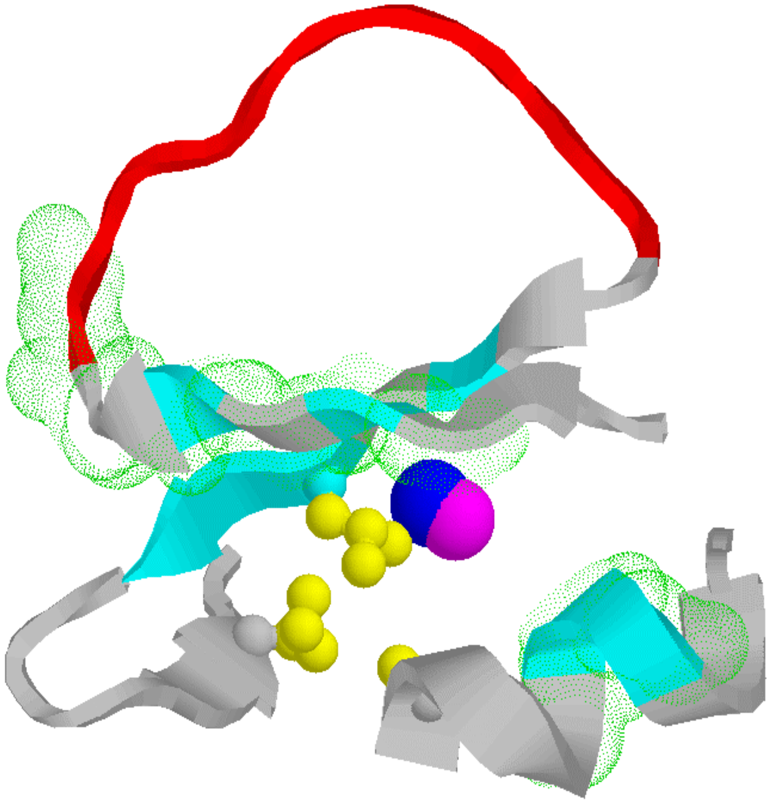

FIGURE 1C

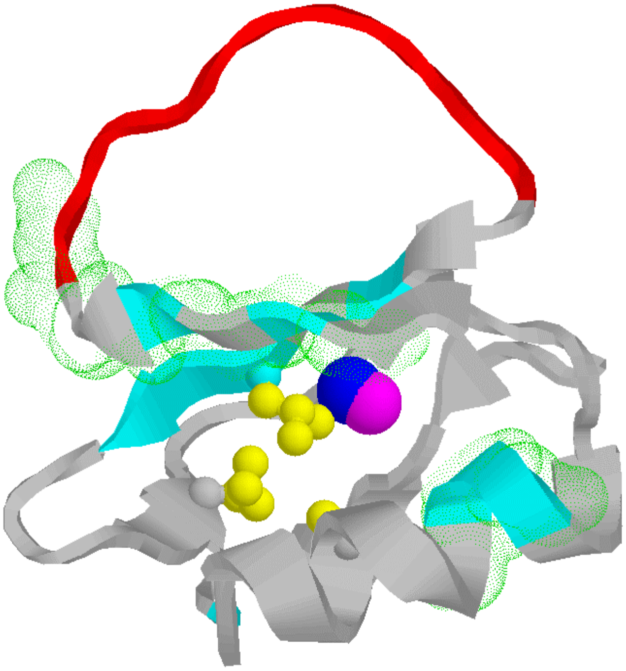

FIGURE 1D

**TABLE 1**
**Residues Around the Positive and Negative Charge Centers (CC+, CC-), of the Hydrogen Exchange Core (HX) and of the Folding Nucleus (NMR), CI2 from Barley (PDB 1 ciq)**

| CC+ | CC- | HX[44] | NMR[42] |
|---|---|---|---|
| W5* | W5 | | |
| L8* | L8 | | |
| A16* | A16 | — | **A16** |
| I20* | I20 | **I20** | |
| — | L21_ | **L21** | |
| I29_ | I29 | | |
| I30_ | I30 | **I30** | |
| — | — | **L32_** | |
| V47* | V47 | **V47** | |
| L49* | L49 | **L49** | **L49** |
| F50* | F50 | **F50** | |
| I57* | I57 | — | **I57** |
| P61* | P61 | | |

*Note*: Only hydrophobic residues are listed. Conserved residues are marked with *. Residues in this table comprise less than 1/3 of all hydrophobic residues in CI2.

## CONCLUSIONS AND PERSPECTIVES

Thermodynamic analyses successfully match the results of the experimental studies of protein folding for a number of proteins. The standard hydrophobic core model provides a qualitative description of the protein structure and can be used for modeling. Such a description, however, does not explicitly treat any of the basic physical forces (in particular, long-range electrostatic interactions). Folding is also a rapid process that occurs on millisecond and microsecond time scales. The thermodynamic descriptions, being inherently statistical, cannot provide a clear physical base for a rapid formation of a highly specific architecture of a protein in such a short time. At the same time, incorporating electrostatic interactions even on a simplistic level (such as in a charge center model, for example) as well as not overestimating the significance of the hydrophobic collapse (such as in the models of local hydrophobic collapse) at least provides a qualitative description for both of the rapidness and the specificity of the protein folding. In other words, the folding is considered as the result of the dynamic balance (cooperation) between the electrostatic and the Van Der Waals forces. Such simple but qualitatively accurate physical models not only allow us to formulate folding scenarios that correlate well with the experimental data, but also can be used for an effective identification of the folding cores and folding nuclei solely from a spatial structure of a protein.

## ACKNOWLEDGMENT

The work was supported in part by the NIH grants CA 76259 and GM 62920. We thank Dr. I. Weber for corrections of English grammar and for helpful remarks on the manuscript.

## BIOSKETCH

**Ivan Y. Torshin** received his Ph.D. in Physical Chemistry from Moscow State University and currently holds a Research Associate/Faculty position at Georgia State University in Atlanta. His research interests include protein folding, folding nucleus, protein structure prediction, bioinformatics, biophysics, molecular mechanics, molecular simulations, biochemistry, immunology, sequence analysis, databases, prediction of functional properties of proteins, ribozymes, nucleic acid binding proteins, enzymatic catalysis, protein association/dissociation, protein-protein interactions, etc.
